# Controlled Polymerization

**DOI:** 10.3390/polym15061379

**Published:** 2023-03-10

**Authors:** Pavel V. Ivchenko

**Affiliations:** A.V. Topchiev Institute of Petrochemical Synthesis RAS, 29 Leninsky Pr., 119991 Moscow, Russia; phpasha1@yandex.ru

An amazing variety of polymerization mechanisms enables the creation of polymers with given microstructures and comonomer sequences. The term ‘controlled polymerization’ often refers to free-radical reactions in which chain termination is suppressed through the introduction of the reagents forming ‘dormant sites’ with ‘living’ polymer chains. Atom transfer radical polymerization (ATRP), reversible addition/fragmentation chain transfer polymerization (RAFT), and nitroxide-mediated polymerization (NMP) are common examples of controlled polymerization processes. However, it is reasonable to go beyond this conventional meaning. Controlled polymerization refers to the formulation of a polymer microstructure in a predetermined number and sequence of comonomer units, no matter how it is caused. The concept of the “control” can be widened, for example, via mutual comonomer reactivity (sequence-controlled polymerization), through the use of cross-coupling reactions (the synthesis of conjugated polymers), ‘living’ catalytic ring-opening polymerization, etc.

This Special Issue includes ten articles that highlight advances in controlled polymerization in the widest sense of the word. Despite the relatively small number of articles, these studies adopt qualitatively different approaches to polymers with a given composition, microstructure and functionality (Figure 1). Below is a brief summary of the papers included in this issue, considering the different approaches to the synthesis of macromolecules with given comonomer sequences, designed for different applications.

The study of Joumaa et al. [1] focuses on the RAFT-based development of complex copolymers containing methacrylic acid acrylate (MAA), poly(ethylene oxide) methyl ether methacrylate (PEOMA), styrene and new monomer N-(4-styrylmethyl)-4,5-bis(diphenylphosphino)phenoxazine, which involves polymerization-induced self-assembly (PISA) in the second step and chain cross-linking with di(ethylene glycol) dimethacrylate (DEGDMA) in the final step (Figure 1). The polymer particles, loaded with a [Rh(acac)(CO)_2_] precatalyst, yield Rh-nixantphos@CCM and were used as catalytic nanoreactors for the aqueous biphasic hydroformylation of oct-1-ene. These catalysts demonstrated a high regioselectivity (with nonanal formation), but moderate activity, attributed to limitations of mass transport.

The controlled synthesis of the random copolymer of pentafluorostyrene (PFS) and methyl methacrylate (MMA) with different formulations and an average molecular weight of (*M*_n_) ~25 kDa was conducted by Peltekoff et al. using NMP [2]. A number of copolymers with molar contents of FPS 0.08–1.00, *M*_n_ 10.5–51.7 kDa and *Ð*_M_ 1.13–1.42 were prepared and employed in the fabrication of metal–insulator–metal capacitors. The dielectric constant of the copolymers linearly decreased with increasing PFS content. Bottom-gate, top-contact copper phthalocyanine (CuPc)-based organic thin-film transistors were also fabricated using these random copolymers as a dielectric layer. A reduction in threshold voltage with increasing fluorine content was detected, but materials that were too rich in fluorine content had poor semiconductor adhesion.

In [3], Motoyanagi, Oguri and Minoda reported the controlled synthesis of alternating copolymers via the copolymerization of 2-(2-(vinyloxy)ethoxy)ethanol (DEGV) and N-ethylmaleimide (EtMI) with and without 2-cyano-2-propyl benzodithioate (CPDB) as a RAFT agent. The content of EtMI was 51–57 mol% (^1^H NMR data), and copolymer composition was weakly dependent on the reaction time and *M*_n_, thus confirming the alternating microstructure of the product. Copolymers were found to be soluble in cold water (0–5 °C) and had a similar behavior to lower critical solution temperature (LCST)-type thermoresponsive materials. Phase separation was detected in the interval *T*_cp_ of 15–35 °C, depending on the molecular weight characteristics of poly(EtMI-*alt*-DEGV).

The first article of the Nifant’ev group [4], published in this Special Issue, is devoted to the development of catalytic approaches to random copolymers of *L*-lactide (*L*-LA) and ε-caprolactone (εCL). BnOH-activated complexes, (BHT)Mg(THF)_2_^n^Bu, (BHT)_2_AlMe and [(BHT)ZnEt]_2_, based on 2,6-di-*tert*-butyl-4-methylphenol (BHT-H), demonstrated very different catalytic behavior in the copolymerization of *L*-LA and εCL. Therefore, even at a 1:5 *L*-LA/εCL ratio, the Mg complex catalyzed the homopolymerization of *L*-LA without co-ROP of εCL. On the contrary, the Zn complex efficiently catalyzed random *L*-LA/εCL copolymerization; the presence of mono-lactate subunits in the copolymer clearly verified the transesterification mechanism of copolymer formation, and the Al complex was less active. Epimerization and transesterification processes were analyzed using the density functional theory (DFT) modeling, which explains the qualitative difference in catalytic behavior between Mg and Zn complexes via the difference in the preferred types of metal coordination on the PLA chain (*k*^2^ and *k*^3^, respectively). The transesterification of commercially available poly(*L*-LA) via εCL created a highly statistical copolymer, mainly containing mono-lactate fragments. The applicability of the Zn complex in the synthesis of random copolymers of εCL with methyl glycolide, ethyl ethylene phosphonate, and ethyl ethylene phosphate was also featured in this study.

The control of the microstructure and, consequently, the properties of lignin-based polyols (LBPs) was demonstrated by Perez-Arce et al., who utilized lignin, a by-product of the pulp industry, via the ‘organosolv lignin’ (OL)-initiated cationic ROP of oxiranes and THF [5]. BF_3_∙Et_2_O was used as a ROP catalyst, THF served as a monomer and solvent. By varying the butylene oxide (BO)/OL ratio, reaction temperature, and BO addition flow rate, these authors have been able to control comonomer ratio and content. A number of oxiranes, including 2-(but-3-en-1-yl)oxirane, glycidol and epichlorohydrin, were also introduced in ROP when polyols were obtained.

The second article of the Nifant’ev group presented in this Special Issue [6] reveals another aspect of the coordination ROP of cyclic esters and cyclic phosphoesters, chain-end functionalization via the termination of ‘living’ coordination ROP of εCL, *L*-LA, ethyl ethylene phosphonate (EtEP) or ethyl ethylene phosphate by acyl chlorides containing oxysuccinimide (OS) or maleimide (MI) fragments. At least a 65% degree of functionalization were achieved, and the yields of poly(εCL)-OS, poly(*L*-LA)-MI and poly(EtEP)-MI were almost quantitative. The suggested synthetic approach to OS- and MI-functionalized polymers allows the synthesis of high-molecular-weight products (*DP*_n_ = 110–170) based on lactones, lactides, cyclic phosphonates and cyclic phosphates, which are able to bind with amines and thiols, thereby uncovering broad perspectives for the biomedical applications of these materials.

Controlling the polymerization of supramolecular self-assembly through external stimuli was explored by Gregorić et al. via the influences of bis(amino acid vinyl ester) fumaramide self-assemblies on γ-ray- and UV-induced polymerization [7]. These self-assemblies had filamentous structure composed of fibers and fiber bundles with nanometer dimensions entangled or organized in a three-dimensional network that was retained during polymerization.

RAFT polymerization was used by Ruiu et al. [8] for the controlled synthesis of fluorinated (co)polymers bearing metal-complexing groups (triphenylphosphine, acetylacetate, thioacetate and thiol). These copolymers were found to be soluble in supercritical CO_2_, and their use for the extraction of the end-of-life liquid or solid wastes containing metals is reported in forthcoming articles.

Among the manuscripts published in this Special Issue were two review articles. The perspective paper, prepared by Wang, Nguyen and Gildersleeve [9], summarizes the studies (76 references) that adopt different concurrent polymerization or side-chain functionalization approaches with ATRP. This perspective summarizes the different types of concurrent reactions used together with ATRP, namely, ring-opening polymerization (ROP), ring-opening metathesis polymerization (ROMP), RAFT polymerization, atom transfer radical addition (ATRA), Cu/azide click chemistry and other side-group modification processes. The applicability of this approach to the synthesis of block copolymers, brush polymers, networks, and polymers with functional side groups was not only well demonstrated, but the authors also highlighted the main limitations of the method and suggested future research directions in this area.

Abdel Baki, Dib, and Sahin [10] reviewed the main features of the ROP of cyclic carbonates, including kinetic and thermodynamic aspects of the polymerization, different reaction mechanisms and variety of the ROP catalysts (100 references). The review does not overlook the alternative approach to aliphatic polycarbonates based on the catalytic ROP of oxiranes in the presence of CO_2_.

As the editor of this Special Issue, I note that the concept of the control of polymerization was implemented by researchers from Canada, Croatia, Denmark, France, Germany, Japan, Kuwait, Romania, Russia, Spain, and the United States in a variety of ways in multidisciplinary research fields. I hope that this collection will encourage interest in this research field.

## Figures and Tables

**Figure 1:**
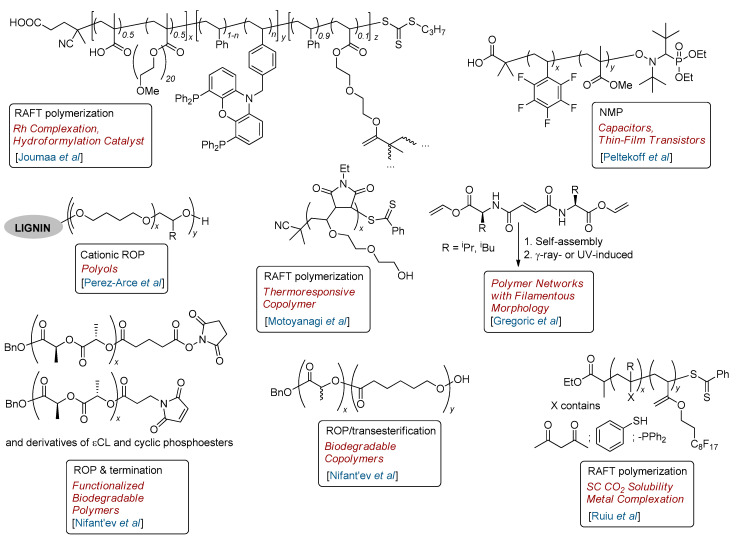
Special Issue entitled ‘Controlled Polymerization’: copolymers synthesized in a targeted and controlled manner [1,2,3,4,5,6,7,8].
